# Erie County Medical Society

**Published:** 1856-09

**Authors:** 


*Erie County Medical Society.*—During the past month, the members of this society have been in a painful state of suspense. Rumors of war, of desperate lawsuits, of fast-coming ruin, have been rife. Expelled members have been big with threats. They, with a lean corporal's guard of sympathizers, have talked loudly of mandamus and indictment. Their happy faces have been seen around the doors of the grand-jury room, but as yet no result obtains. Gentlemen rebels, please to hurry up the war! For two years we have watched the brewing storm, until we fear the process of fermentation has resulted in stale vinegar only.

